# Behaviour change techniques taxonomy v1: Feedback to inform the development of an ontology

**DOI:** 10.12688/wellcomeopenres.18002.1

**Published:** 2022-08-12

**Authors:** Elizabeth Corker, Marta M. Marques, Marie Johnston, Robert West, Janna Hastings, Susan Michie

**Affiliations:** 1 Centre for Behaviour Change, University College London, Lisbon, UK; 2 Clinical and Applied Psychology Unit, Department of Psychology, University of Sheffield, Sheffield, UK; 3 Comprehensive Health Research Centre (CHRC), NOVA Medical School|Faculdade de Ciências Médicas (NMS|FCM,) Universidade Nova de Lisboa, Lisboa, Portugal., Portugal; 4 Aberdeen Health Psychology Group, University of Aberdeen, Aberdeen, UK

**Keywords:** behaviour change techniques, taxonomy, ontology, user feedback, intervention reporting

## Abstract

**Background:** To build cumulative evidence about what works in behaviour change interventions, efforts have been made to develop classification systems for specifying the content of interventions. The Behaviour Change Techniques (BCT) Taxonomy v1 (BCTTv1) is one of the most widely used classifications of behaviour change techniques across a variety of behaviours. The BCTTv1 was intentionally named version 1 to allow for further revisions to the taxonomy. This study aimed to gather data to inform the development of BCTTv1 into a more elaborated knowledge structure, an ontology.

**Methods:** Feedback from users of BCTTv1 about limitations and proposed improvements was collected through the BCT website, a user survey, researchers and experts involved in the Human Behaviour Change Project, and a consultation. In addition, relevant published research reports and other classification systems of BCTs were analysed. These data were synthesised to produce recommendations to inform the development of an ontology of BCTs.

**Results:** A total of 282 comments from six sources were reviewed and synthesised into four categories of suggestions: additional BCTs, amendments to labels and definitions of specific BCTs, amendments to the groupings, and general improvements. Feedback suggested some lack of clarity regarding understanding and identifying techniques from labels, definitions, and examples; distinctions and relations between BCTs; and knowing what they would look like in practice. Three recommendations resulted from this analysis: to review the label and definition of each BCT, the 16 groupings of BCTs, and the examples illustrating BCTs.

**Conclusions** *:* This review of feedback about BCTTv1 identified the need to improve the precision and knowledge structure of the current taxonomy. A BCT ontology would enable the specification of relationships between BCTs, more precise definitions, and allow better interoperability with other ontologies. This ontology will be developed as part of the Human Behaviour Change Project.

## Introduction

To build cumulative evidence about ‘what works’ for behaviour change interventions aiming to influence human behaviours, efforts have been made to develop classification systems for specifying the content of interventions. Examples include behaviour change techniques (BCTs), defined as planned processes that are the smallest parts of the content of a behaviour change intervention that are observable, replicable and on their own have the potential to bring about behaviour change ( Michie *et al.,* 2021). These classifications provide a standardized way and common language to describe BCTs, contributing to the improvement of intervention reports and evidence syntheses, and as a result, to the implementation of effective behaviour change interventions in research and practical settings.

The Behaviour Change Techniques Taxonomy v1 (BCTT v1) ( Michie *et al.,* 2013) is the most widely used classification of BCTs. The BCTTv1 provides a list of 93 clearly labelled and defined BCTs, organised in 16 higher-order groupings. The BCTTv1 was developed over an iterative programme of research studies. This involved identifying commonly used techniques in interventions across various health behaviours, labelling and description of distinct and non-overlapping techniques, consultation with experts for feedback on the BCTs, development of a hierarchical structure, and validation of the BCTTv1 through coding intervention reports ( Michie *et al.,* 2013; Michie *et al.,* 2015). 400 experts from around the world contributed to the development and validation of BCTTv1. Resources were developed to support the use of BCTTv1, including an app ( www.ucl.ac.uk/health-psychology/bcttaxonomy/BCT_app1), a database of studies of interventions coded using BCTTv1 ( www.bct-taxonomy.com/interventions), and online training to guide the identification of BCTs in published papers ( www.bct-taxonomy.com/).

The BCTTv1 has been widely adopted, tested, and applied internationally (e.g., >1400 people from 33 countries/13 low- and middle-income countries (LMIC)s have participated in the BCTTv1 training; 4,830 citations of the main BCTTv1 papers ( Michie *et al.,* 2013; Michie *et al.,* 2015)). The BCTTv1 has been mainly used to identify, through systematic reviews and meta-analysis, the presence of individual BCTs and groups of BCTs that are more frequently used and/or more effective across a wide range of behaviour change interventions in diverse populations ( West *et al.,* 2020). It has also been used to inform intervention design and evaluation, frequently through its integration in the Behaviour Change Wheel Framework ( Michie *et al.,* 2011).

The BCTTv1 was named ‘version 1’ to signal that further revisions would be expected based on; 1) emerging evidence, 2) feedback from users for updating, advancing, and increasing the scientific and practical value of BCTTv1 (e.g., additional BCTs, structural changes), and 3) new knowledge on alternative improved classification methods.

Ontologies offer a more comprehensive and expressive way of representing information than taxonomies ( Hastings, 2017). A comprehensive Behaviour Change Intervention Ontology (BCIO) is being developed as part of the Human Behaviour-Change ( Michie *et al.*, 2017; Michie *et al.,* 2021). The BCIO consists of an upper level with 42 entities, specifying features of behaviour change interventions, such as mode of delivery ( Marques *et al.,* 2021), source ( Norris *et al.,* 2021), and setting ( Norris *et al.,* 2020). One of the entities in the BCIO is BCT *,* specified as part of the content in a given behaviour change intervention scenario ( Michie *et al.,* 2021).

Ontologies are inter-operable, which means that an ontology of BCTs can be linked to the other entities specified in the BCIO as well as to other relevant social, behavioural, and interventions ontologies, allowing integration of evidence across disciplinary and topic domains. This enables the answering of questions about the effectiveness of behaviour change interventions and how effects are modified according to different behaviours and contexts (populations and settings), and about the way components of intervention work together to achieve behaviour change. As well as advancing understanding about variation in effects across interventions, an ontology of behaviour change interventions can advance understanding of processes of change, i.e., their mechanisms of action. As ontologies are computer-readable, they can be used to synthesise large amounts of data using artificial-intelligence based methods to provide evidence-based knowledge on the components of behaviour change interventions that are more effective and how they relate with each other.

The current study aimed to gather data with which to update the BCTTv1 and convert it into a BCT ontology. This paper reports an analysis of feedback about BCTTv1 from experts and intervention developers from a variety of fields. Feedback was sought on the limitations and improvements that could be made to BCTTv1 in relation to adding BCTs, improving BCT labels and definitions, and changing groupings and structure. Recommendations are made based on the feedback.

## Methods

### Ethical statement

Ethical approval was granted by University College London’s ethics committee (CEHP/2016/555). Informed consent was obtained from participants prior to participation in the surveys that were conducted as part of this study, by indicating in the surveys if their answers could be published

### Design and sample

This study consisted of three stages: 1) seeking feedback from users of the BCTTv1, 2) synthesising feedback, and 3) producing recommendations relating to improvements to classification of BCTs. Participant consent was gained for each source of feedback.

### Stage one: Gathering feedback about the BCTTv1

Feedback about the limitations and proposed improvements to the BCTTv1 was sought from several sources: 1) researchers from the Human Behaviour-Change Project who coded 512 papers using the BCTTv1; 2) data from the Theory and Techniques Project expert consensus exercise ( Connell *et al.,* 2019); 3) two online surveys designed to gather feedback regarding BCTs; 4). a consultation exercise with users of BCTs, including researchers and implementers; 5) relevant published research reports proposing new BCTs and/or changes to BCTTv1 or other classification systems of BCTs. The online surveys were conducted using Qualtrics. The first survey was open to any use of the BCTTv1 wishing to provide feedback about BCTs that were not included in the BCTTv1, amendments to BCTs, BCTs that were difficult to use, adaptations and translations of the BCTTv1, data on reliability, and general suggestions for improvements. The survey contained closed and open-ended questions. Recruitment was conducted through advertising the link to the survey in social media, and Centre for Behaviour Change Newsletters. The second survey inquired about the use of the BCTTv1 (reason for using, research questions addressed when using the BCTTv1) and general improvements to be made to the BCTTv1. Recruitment was conducted via email, contacting BCTTv1 users who had previously signed up to a list of stakeholders for the Human Behaviour Change Project. Details for each source of feedback are summarised in Table 1 and details for each paper reviewed are summarised in Table 2.

**Table 1.** Sources of Behaviour Change Techniques Taxonomy v1 (BCTTv1) feedback.

Feedback source	Type and treatment of data	Year data collected and reference/ website
1. Human Behaviour- Change Project	Qualitative analysis of documents related to the annotations of BCTs in intervention reports using the BCTTv1. Research activities within the Human Behaviour Change Project relating to the development of the Behaviour Change Intervention Ontology have included keeping notes relating to the use of BCTs and the BCTTv1.	2017–2018 https://www.humanbehaviourchange.org/
2. Theory and Techniques Project ( Connell *et al.,* 2019)	Secondary analysis of qualitative data relating to the Theory and Techniques project. As part of the Theories and Techniques project 105 behaviour science experts provided comments regarding BCTs.	2015
3. BCTTv1 online feedback portal ( West *et al.,* 2020)	Qualitative analysis of data collected from BCTTv1 users through an open online portal. A portal on the BCTs Taxonomy v1 website allowed users to submit comments on the BCTs and the BCTTv1.	2015–2020
4. Consultation report ( West *et al.,* 2020)	Secondary analysis of qualitative data relating to use of the BCTs Taxonomy v1. A consultation exercise was completed, during which participants provided comments relating to their use of BCTs or the BCTTv1.	2019
5. BCTTv1 user survey ( West *et al.,* 2020)	Qualitative analysis of data collected from BCTTv1 users. Researchers and behaviour scientists completed a survey designed to provide feedback regarding their use of BCTs or the BCTTv1. This survey was conducted to gather additional feedback to what was collected through the online feedback portal.	2021
6. Reports of behaviour classification systems or BCTs	Secondary analysis of qualitative data related to BCTs or the BCTTv1. Several research reports have been published that outline behaviour classification systems or give direct recommendations for revisions to specific BCTs. Relevant research reports were identified by: • participants in the feedback exercises • correspondence sent to the research team • a forward citation search, conducted in 2021, from the BCTTv1 development, published in 2013.	2022

**Table 2.** Papers initially reviewed.

Behaviour classification system related paper	Source of identification	Inclusion in analysis
Towards a taxonomy of behaviour change techniques for promoting shared decision making ( Agbadjé *et al.,* 2020	Forward citation search	Yes
MINDSPACE ( Dolan *et al.,* 2010)	Suggested by research team and identified by survey participant, consultation	Yes
A systematic review of recruitment strategies and BCTs in group-based diabetes prevention programmes focusing on uptake and retention ( Begum *et al.,* 2020)	Sent to research team and forward citation search	Yes
BCTs associated with smoking cessation in intervention and comparator groups of randomized controlled trials: a systematic review and meta-regression ( Black *et al.,* 2020)	Identified by HBCP team and forward citation search	Yes
Social norms interventions to change clinical behaviour in health workers: a systematic review and meta-analysis ( Cotterill *et al.,* 2020)	Identified by survey participant, and forward citation search	Yes
Assessing and promoting the use of implementation intentions in clinical practice ( Duhne *et al.,* 2020)	Identified by survey participant, and forward citation search	Yes
Identifying content-based and relational techniques to change behaviour in motivational interviewing ( Hardcastle *et al.,* 2017)	Identified by survey participant, and portal and forward citation search	Yes
The TIPPME intervention typology for changing environments to change behaviour ( Hollands *et al.,* 2017)	Identified by survey participant, in TaT project and forward citation search	Yes
The compendium of self-enactable techniques to change and self-manage motivation and behaviour v.1.0 ( Knittle *et al.,* 2020)	Identified by survey participant portal	Yes
A taxonomy of behaviour change methods: an Intervention Mapping approach ( Kok *et al.,* 2016)	Suggested by research team	Yes
Social prescribing and behaviour change: proposal of a new behaviour change technique concerning the ‘connection’ step ( Cunningham *et al.,* 2022)	Sent to research team and forward citation search	Yes
EAST Four simple ways to apply behavioural insights ( The Behavioural Insights Team, 2014)	Suggested by research team and identified by survey participant, consultation	No
Everything should be as simple as possible, but no simpler: towards a protocol for accumulating evidence regarding the active content of health behaviour change interventions ( Peters *et al.,* 2015)	Identified by survey participant user survey	No

### Stage two: Synthesising feedback

Data from each source of feedback relating to the BCTTv1 were reviewed by two authors (EC and MM). The feedback from BCTTv1 users and the recommendations made for revisions within published papers were added to four broad categories of feedback were identified that captured the main issues raised: additional BCTs, amendments to labels or definitions of BCTs, amendments to the BCT groupings, and general improvements. Data were synthesised into a single document, enabling examination across each data source (see underlying data - full extraction data) ( West *et al.,* 2020). Authors then discuss each recommendation and how they could be addressed. Suggestions to change any aspects of the BCTTv1 were then discussed with the core research team. The outputs from the initial review were then discussed and revised by two authors (EC and MJ).

### Stage three: Producing recommendations on developing BCTTv1 into a BCT ontology

Five behavioural science experts (EC, MM, MJ, SM, and RW) and one ontology expert (JH) reviewed each comment in the output from Stage 2 and drafted and then refined recommended actions relating to each piece of feedback. In addition to the synthesised feedback, each BCT label and definition was reviewed to ensure that they were consistent with good ontological practice ( Michie *et al.,* 2019) and that each label is aligned with its definition ( Michie *et al.,* 2021), that is, each BCT should be: 1) a planned process, 2) the smallest part of an intervention that on its own can bring about change in behaviour 3) observable, 4) replicable, and 5) have the potential to bring about behaviour change. A list of recommendations relating to the labels and definitions of BCTs, along with the structure, hierarchy and relationships was produced.

## Results

### Stage one: Feedback about BCTTv1

Feedback was gathered from several sources: 1) researchers from the Human Behaviour-Change Project (n=4 researchers; 512 papers coded for BCTs); 2) data from the Theory and Techniques Project expert consensus exercise (n=105); 3) two online surveys designed to gather feedback regarding BCTs (BCTTv1 online portal n=27 and BCTTv1 user survey n=68); 4) a consultation exercise with users of BCTs, including researchers and implementers (n=22); 5) relevant published research reports proposing new BCTs and/or changes to BCTTv1 or other classification systems of BCTs (n=11).

The number of users that contributed to each feedback source ranged from 22 to 105. The forward citation search identified 2,562 research reports. In eight of these reports, a behaviour classification system, recommendations for revisions to specific BCTs, or recommendations for revisions to the BCTTv1 were identified ( Table 2). Seven of these were also identified by either a participant in the user feedback exercises or the research team. A further three research reports were suggested by the research team and two more were suggested by a participant in the user feedback exercises, giving a total of 13 reports. Two were not taken forward to the data analysis process because they described general ways of thinking about behaviour rather than BCTs.

### Stage two: Synthesis of feedback

A total of 438 comments from the feedback exercises and published reports were reviewed. Table 3 reports the number of comments reviewed from each feedback source and Table 4 reports the numbers of comments removed from the analysis. During an initial screening process, 156 comments were removed from the analysis. Reasons for removing comments were:

- the comment contained a suggestion that was deemed to be beyond the scope of the development of BCTs
- the suggestion made had already been incorporated into other Behaviour Change Intervention Ontology work
- a specific suggestion was not made
- the suggestion did not fit with the study definition of behaviour or of behaviour change technique.

**Table 3.** Number of suggestions from each source of feedback.

Feedback source	Total number of suggestions
Theory and Techniques project (n=105)	35
Human Behaviour Change Project	31
BCTTv1 * user portal (n= 27)	71
BCTTv1 * user survey (n= 68)	123
Consultation report (n =22)	50
Reports of behaviour classification systems or behaviour change techniques (BCTs) (n=10)	128
**Total number of suggestions**	**438**

* Behaviour Change Techniques Taxonomy v1

**Table 4.** Number of comments removed from analysis.

Reason for removal of comment	N
Suggestion beyond the scope of the behaviour change techniques (BCTs)	65
The suggestion made had already been incorporated into other Behaviour Change Intervention Ontology work	10
No specific suggestion made	61
The suggestion did not fit with the definition of behaviour or the definition of a BCT	20
**Total number of comments removed from analysis**	**156**

The remaining 282 comments were reviewed and sorted into four categories of suggestion: additional BCTs (n=32), amendments to the labels or definitions of BCTs (n=92), amendments to BCT groupings (n=9), and general improvements (n=17). These numbers do not equal the total number of comments as some comments were sorted into more than one category, and several comments contained the same suggestion. ( West *et al.,* 2020)

### Additional BCTs

32 comments were made, containing 47 suggestions for new BCTs. A further 22 BCTs were considered by participants to be more than one technique. Review by the research team resulted in 22 suggestions for new BCTs, to be further developed in an ontology of BCTs.

### Labels or definitions of BCTs

92 comments contained suggestions relating to amending a BCT label, definition or example. The number of suggestions made per BCT ranged from 0–11 ( Table 5). Based on these comments, the research team developed recommendations for revision of labels and definitions for each BCT, taking into consideration ontological best practice ( Michie *et al.,* 2019; Michie *et al.,* 2021). Amendments should be made to all BCT labels to ensure that each label is clearly aligned to a specific BCT definition. Additionally, in accordance with ontological best practice, BCTs that refer to two separate techniques within the same definition should be separated out, for example, ‘problem solving’ should be ‘problem solving BCT’ and ‘prompt problem solving BCT’. Brackets should also be removed from BCT labels, for example, the label ‘goal setting (behaviour)’ should be ‘goal setting BCT’. In addition, the beginning of each BCT definition should be amended to ensure that the definition is clearly aligned to a specific grouping, for example, the research team proposes that the start of the definition for ‘goal setting BCT’ is ‘a goal directed BCT that changes behaviour by….’, where ‘goal directed BCT’ is the label for the specific group in which ‘goal setting BCT’ is placed.

**Table 5.** Number of suggestions made relating to specific behaviour change techniques (BCTs).

BCTTv1 * no.	BCTTv1 label	N of comments
1.1	Goal setting (behaviour)	6
1.2	Problem Solving	4
1.3	Goal setting (outcome)	2
1.4	Action planning	6
1.5	Review behaviour goal(s)	0
1.6	Discrepancy between current behaviour and goal	2
1.7	Review outcome goal(s)	0
1.8	Behavioural contract	1
1.9	Commitment	2
2.1	Monitoring of behaviour by others without feedback	1
2.2	Feedback on behaviour	1
2.3	Self-monitoring of behaviour	2
2.4	Self-monitoring of outcome(s) of behaviour	1
2.5	Monitoring outcome(s) of behaviour by others without feedback	0
2.6	Biofeedback	0
2.7	Feedback on outcome(s) of behaviour	1
3.1	Social support (unspecified)	11
3.2	Social support (practical)	1
3.3	Social support (emotional)	2
4.1	Instruction on how to perform a behaviour	3
4.2	Information about antecedents	2
4.3	Re-attribution	1
4.4	Behavioural experiments	0
5.1	Information about health consequences	1
5.2	Salience of consequences	3
5.3	Information about social and environmental consequences	0
5.4	Monitoring of emotional consequences	1
5.5	Anticipated regret	2
5.6	Information about emotional consequences	2
6.1	Demonstration of the behaviour	2
6.2	Social comparison	2
6.3	Information about others’ approval	3
7.1	Prompts/cues	1
7.2	Cue signalling reward	1
7.3	Reduce prompts/cues	0
7.4	Remove access to the reward	0
7.5	Remove aversive stimulus	0
7.6	Satiation	0
7.7	Exposure	1
7.8	Associative learning	1
8.1	Behavioural practice/ rehearsal	4
8.2	Behaviour substitution	0
8.3	Habit formation	0
8.4	Habit reversal	1
8.5	Overcorrection	0
8.6	Generalisation of a target behaviour	0
8.7	Graded tasks	5
9.1	Credible source	6
9.2	Pros and cons	2
9.3	Comparative imagining of future outcomes	1
10.1	Material incentive (behaviour)	1
10.2	Material reward (behaviour)	5
10.3	Non-specific reward	2
10.4	Social reward	1
10.5	Social incentive	1
10.6	Non-specific incentive	2
10.7	Self-incentive	3
10.8	Incentive (outcome)	1
10.9	Self-reward	5
10.10	Reward (outcome)	0
10.11	Future punishment	4
11.1	Pharmacological support	4
11.2	Reduce negative emotions	4
11.3	Conserving mental resources	0
11.4	Paradoxical instructions	0
12.1	Restructuring the physical environment	7
12.2	Restructuring the social environment	4
12.3	Avoidance/reducing exposure to cues for the behaviour	2
12.4	Distraction	1
12.5	Adding objects to the environment	4
12.6	Body changes	3
13.1	Identification of self as role model	0
13.2	Framing/reframing	1
13.3	Incompatible beliefs	0
13.4	Valued self-identity	5
13.5	Identity associated with changed behaviour	2
14.1	Behaviour cost	1
14.2	Punishment	2
14.3	Remove reward	1
14.4	Reward approximation	0
14.5	Rewarding completion	0
14.6	Situation-specific reward	0
14.7	Reward incompatible behaviour	1
14.8	Reward alternative behaviour	1
14.9	Reduce reward frequency	1
14.10	Remove punishment	0
15.1	Verbal persuasion about capability	3
15.2	Mental rehearsal of successful performance	0
15.3	Focus on past success	1
16.1	Imaginary punishment	1
16.2	Imaginary reward	0
16.3	Vicarious consequences	2

*Behaviour Change Techniques Taxonomy v1

### Amendments to BCTTv1 groupings

Nine comments were made in relation to the 16 BCTTv1 groupings, with each grouping attracting up to four comments ( Table 6). Content of the feedback consisted of requests for clarification of group definitions and creations of new groups. This feedback reinforced the need to review the original BCT groupings, taking into consideration ontological principals.

**Table 6.** Number of suggestions made relating to each grouping of behaviour change techniques (BCTs).

BCTTv1 * grouping no.	BCTTv1 grouping label	N of comments
1.	Goals and planning	1
2.	Feedback and monitoring	1
3.	Social support	0
4.	Shaping knowledge	0
5.	Natural consequences	2
6.	Comparison of behaviour	1
7.	Associations	0
8.	Repetition and substitution	0
9.	Comparison of outcomes	0
10.	Reward and threat	4
11.	Regulation	0
12.	Antecedents	0
13.	Identity	0
14.	Scheduled consequences	0
15.	Self-belief	0
16.	Covert learning	0

*Behaviour Change Techniques Taxonomy v1

### Frequently reported issues

The issue reported most frequently was a lack of clarity of BCT labels and definitions (121 comments). Examples are (for full details, please see West *et al.,* 2020).

“Clarify that this is more specific than the everyday use of the term by enhancing the 'what it is not” (1.4 Action planning)

“Clarify definition to include rewards from participants 'naturally' or integrated in interventions by design” (10.2 Material reward (behaviour))

“Needs more specificity to avoid being a 'catch-all'” (3.1 Social support (unspecified)). 

14 comments referred to difficulties in distinguishing between BCTs, for example

“Better differentiation needed between 10.7 self-incentive and 10.9 self-reward labels” (10.7 Self incentive).

### Stage three: Recommendations for ontology development

Based on Stage 2, three recommendations were made for the next stage of developing the BCT ontology.

1. Review the label and definition of each BCT to ensure that they are consistent with good ontological practice and that each label is aligned with its definition ( Michie *et al.,* 2019; Michie *et al.,* 2021),

2. Review each of the 16 groupings that contain BCTs. Each grouping should be inclusive, that is, the grouping should capture each relevant BCT, as well as exclusive, that is, the grouping should not describe aspects of BCTs that appear in other groupings.

3. Review the examples that are given to illustrate BCTs. Examples of BCTs should span behavioural domains and illustrate only the BCT it is an example of.

## Discussion

The widespread international use of BCTs through systematic reviews and meta-analysis, along with intervention design, implementation and evaluation ( Armitage *et al.,* 2021) demonstrates the utility and need for a BCT classification system. The feedback synthesis and review benefited from the use of six sources of feedback generating almost 300 comments but was inevitably constrained in scope by study resources. The study of expert user views found that the BCTTv1 classification system could be improved. A total of 282 comments were reviewed and synthesised into four categories of feedback producing three recommendations for future development. These were to review the label and definition of each BCT, the BCT groupings, and the examples to illustrate BCTs. 

This review of feedback about BCTTv1 identified the need to improve the precision and knowledge structure of the current taxonomy. The recommendations from this review and synthesis of extensive feedback relating to BCTs will enable a shared understanding of how best to conceptualise and organise BCTs in relation to each other.

From the revision of the BCTTv1 it became clear that this classification would benefit from an ontological structure, enabling clearer internal relationships between different BCTs, as well as relationships between BCTs and other aspects of behaviour change interventions such as mechanisms of action. 

These findings will serve as the basis of developing BCTTv1 into a BCT Ontology. In addition to allowing specification of relationships within the ontology and interoperability with other ontologies, this transformation will also support the future sustainability of the classification: as ontology groupings are based on logical relationships between entities, development of a BCT Ontology will allow for subsequently identified entities to be added where they fit logically, without disrupting previously specified relationships. It will also allow for integration of BCTs within the broader Behaviour Change Intervention Ontology being developed by the Human Behaviour-Change Project ( Michie *et al.,* 2017; Michie *et al.,* 2021), such as source ( Norris *et al.,* 2021), mode of delivery ( Marques *et al.,* 2021), setting ( Norris *et al.,* 2020), and mechanisms of action ( West *et al*., 2020) which in turn will allow annotations and users to access content via a single technical framework and a common set of tools.

By linking with the BCI ontology, the BCT ontology will be a valuable method for investigating the effectiveness of BCTs across contexts, such as populations and settings, and across types of behaviour. It also facilitates the investigation of processes of change, by linking BCTs with their potential mechanisms of action, building on the Theory and Techniques tool ( https://theoryandtechniquetool.humanbehaviourchange.org/). Since ontologies are not static but can be developed to reflect scientific advance, more granular representation or further improvements. Further, a BCT ontology will also allow for continuing development regarding definitions, labels and additional BCTs.

### Limitations

Several limitations should be noted in this research. First, the sample sizes from the survey and open online portal were small, although efforts were made in disseminating these tools within the scientific community. Secondly, the papers proposing changes to BCTs were those the wider research team were familiar with rather than reflecting a systematic literature search. However, we considered this not to be a significant problem given that we drew on a number of diverse sources of feedback. Thirdly, the changes to the BCTs and groupings that were conducted in this study were only reviewed and discuss by the research team, they were not reviewed or tested by BCTTv1 users. An expert consultation activity will be conducted as part of the BCTO development.

## Conclusion

Feedback from users and experts identified a number of ways in which BCTTv1 could be improved including improved labels, definitions and groupings. The analysis of the feedback to the BCTTv1 provides a solid basis for further research development work to create a BCT Ontology that can link up with other ontologies related to behaviour change. This work as a clear practical implication as it identifies the main issues experienced by interventionists using the BCTTv1 and provides clear recommendations for developing a better classification system of BCTs crucial to improve the quality of intervention reporting and evidence synthesis.

## Data availability

### Underlying data

Open Science Framework: Human Behaviour-Change Project > Behavioural Science > Exposure > Intervention > Content > BCTTv1 Feedback paper. https://doi.org/10.17605/OSF.IO/EFP4X ( West *et al.*, 2020)

This project contains the following underlying data:

- Full Extraction data. (file containing the data extracted and analysed from all sources consulted in the study)

### Extended data

Open Science Framework: Human Behaviour-Change Project > Behavioural Science > Exposure > Intervention > Content > BCTTv1 Feedback paper. https://doi.org/10.17605/OSF.IO/EFP4X ( West *et al.,* 2020)

This project contains the following extended data:

- BCTTv1 Online Form. (Questions asked in the Online form made available to anyone wishing to contribute)
- BCTTv1 User Survey (Questions asked in the BCTTv1 User survey)
- UCL BCT Social Enterprise Business Case Report (Report of of informal qualitative research undertaken with users of the BCTTv1)

Data are available under the terms of the Creative Commons Attribution 4.0 International license (CC-BY 4.0)

## References

[ref-2] Agbadjé TT Elidor H Perin MS : Towards a taxonomy of behavior change techniques for promoting shared decision making. *Implement Sci.* 2020;15(1):67. 10.1186/s13012-020-01015-w 32819410 PMC7439658

[ref-3] Armitage CJ Conner M Prestwich A : Investigating which behaviour change techniques work for whom in which contexts delivered by what means: Proposal for an international collaboratory of Centres for Understanding Behaviour Change (CUBiC). *Br J Health Psychol.* 2021;26(1):1–14. 10.1111/bjhp.12479 33080120

[ref-4] Begum S Povey R Ellis N : A systematic review of recruitment strategies and behaviour change techniques in group-based diabetes prevention programmes focusing on uptake and retention. *Diabetes Res Clin Pract.* 2020;166:108273. 10.1016/j.diabres.2020.108273 32590009

[ref-5] Black N Eisma M Viechtbauer W : Variability and Effectiveness of Comparator Group Interventions in Smoking Cessation Trials: A Systematic Review and Meta-Analysis. *Addiction.* 2020;115(9):1607–1617. 10.1111/add.14969 32043675 PMC7496125

[ref-6] Connell LE Carey RN de Bruin M : Links between Behavior Change Techniques and Mechanisms of Action: An Expert Consensus Study. *Ann Behav Med.* 2019;53(8):708–720. 10.1093/abm/kay082 30452535 PMC6636885

[ref-7] Cotterill S Tang MY Powell R : Social norms interventions to change clinical behaviour in health workers: a systematic review and meta-analysis. Southampton (UK): NIHR Journals Library; 2020. 10.3310/hsdr08410 33151653

[ref-13] Cunningham KB Rogowsky RH Carstairs SA : Social prescribing and behaviour change: proposal of a new behaviour change technique concerning the 'connection' step. *Health Psychol Behav Med.* 2022;10(1):121–123. 10.1080/21642850.2021.2019584 35024248 PMC8747515

[ref-8] Dolan P Hallsworth M Halpern D : MINDSPACE: influencing behaviour for public policy. Institute of Government, London, UK. 2010.

[ref-9] Duhne PGS Horan AJ Ross C : Assessing and promoting the use of implementation intentions in clinical practice. *Soc Sci Med.* 2020;265:113490. 10.1016/j.socscimed.2020.113490 33261903

[ref-10] Hardcastle SJ Fortier M Blake N : Identifying content-based and relational techniques to change behaviour in motivational interviewing. *Health Psychol Rev.* 2017;11(1):1–16. 10.1080/17437199.2016.1190659 27189713

[ref-11] Hastings J : Primer on Ontologies. *Methods Mol Biol.* 2017;1446:3–13. 10.1007/978-1-4939-3743-1_1 27812931

[ref-12] Hollands G Bignardi G Johnston M : The TIPPME intervention typology for changing environments to change behaviour. *Nat Hum Behav.* 2017;1:0140. 10.1038/s41562-017-0140

[ref-14] Knittle K Heino M Marques MM : The compendium of self-enactable techniques to change and self-manage motivation and behaviour v.1.0. *Nat Hum Behav.* 2020;4(2):215–223. 10.1038/s41562-019-0798-9 31932687

[ref-15] Kok G Gottlieb NH Peters GJ : A taxonomy of behaviour change methods: an Intervention Mapping approach. *Health Psychol Rev.* 2016;10(3):297–312. 10.1080/17437199.2015.1077155 26262912 PMC4975080

[ref-16] Marques MM Carey RN Norris E : Delivering Behaviour Change Interventions: Development of a Mode of Delivery Ontology [version 2; peer review: 2 approved]. *Wellcome Open Res.* 2021;5:125. 10.12688/wellcomeopenres.15906.2 33824909 PMC7993627

[ref-17] Michie S van Stralen MM West R : The behaviour change wheel: A new method for characterising and designing behaviour change interventions. *Implement Sci.* 2011;6:42. 10.1186/1748-5908-6-42 21513547 PMC3096582

[ref-18] Michie S Richardson M Johnston M : The behavior change technique taxonomy (v1) of 93 hierarchically clustered techniques: Building an international consensus for the reporting of behavior change interventions. *Ann Behav Med.* 2013;46(1):81–95. 10.1007/s12160-013-9486-6 23512568

[ref-19] Michie S Wood CE Johnston M : Behaviour change techniques: The development and evaluation of a taxonomic method for reporting and describing behaviour change interventions (a suite of five studies involving consensus methods, randomised controlled trials and analysis of qualitative data). *Health Technol Assess.* 2015;19(99):1–188. 10.3310/hta19990 26616119 PMC4781650

[ref-20] Michie S Thomas J Johnston M : The Human Behaviour-Change Project: Harnessing the power of Artificial Intelligence and Machine Learning for evidence synthesis and interpretation. *Implement Sci.* 2017;12(1):121. 10.1186/s13012-017-0641-5 29047393 PMC5648456

[ref-21] Michie S West R Finnerty AN : Representation of behaviour change interventions and their evaluation: Development of the Upper Level of the Behaviour Change Intervention Ontology [version 2; peer review: 2 approved]. *Wellcome Open Res.* 2021;5:123. 10.12688/wellcomeopenres.15902.2 33614976 PMC7868854

[ref-22] Michie S West R Hastings J : Creating ontological definitions for use in science. *Qeios.* 2019. 10.32388/ygif9b

[ref-23] Norris E Marques MM Finnerty AN : Development of an Intervention Setting Ontology for behaviour change: Specifying where interventions take place [version 1; peer review: 2 approved]. *Wellcome Open Res.* 2020;5:124. 10.12688/wellcomeopenres.15904.1 32964137 PMC7489274

[ref-24] Norris E Wright AJ Hastings J : Specifying who delivers behaviour change interventions: development of an Intervention Source Ontology [version 1; peer review: 2 approved, 1 approved with reservations]. *Wellcome Open Res.* 2021;6:77. 10.12688/wellcomeopenres.16682.1 34497878 PMC8406443

[ref-25] Peters GJ de Bruin M Crutzen R : Everything should be as simple as possible, but no simpler: towards a protocol for accumulating evidence regarding the active content of health behaviour change interventions. *Health Psychol Rev.* 2015;9(1):1–14. 10.1080/17437199.2013.848409 25793484 PMC4376231

[ref-26] The Behavioural Insights Team: EAST: Four simple ways to apply behavioural insights.2014. Reference Source

[ref-27] West R Michie S Shawe-Taylor J : Human Behaviour-Change Project. Dataset.2020. 10.17605/OSF.IO/UXWDB PMC7287511 32566761

