# Supplementary material for: Behaviour change techniques taxonomy v1: Feedback to inform the development of an ontology
Source: Wellcome Open Res. 2023 Jan 20;7:211. Originally published 2022 Aug 12. [Version 2] doi: 10.12688/wellcomeopenres.18002.2 (PMC10511844; doi:10.12688/wellcomeopenres.18002.2)
Supplement: Peer review history

# Reviewer report: Reviewer response for version 2

**DOI:** 10.21956/wellcomeopenres.20917.r54429

**Published:** 2023-02-06

**Authors:** Stephan Dombrowski

**Affiliations:** 1 Faculty of Kinesiology, University of New Brunswick, Fredericton, NB, Canada

I approve of this updated version.

Is the work clearly and accurately presented and does it cite the current literature?

Yes

If applicable, is the statistical analysis and its interpretation appropriate?

Not applicable

Are all the source data underlying the results available to ensure full reproducibility?

Yes

Is the study design appropriate and is the work technically sound?

Yes

Are the conclusions drawn adequately supported by the results?

Yes

Are sufficient details of methods and analysis provided to allow replication by others?

Partly

Reviewer Expertise:

NA

I confirm that I have read this submission and believe that I have an appropriate level of expertise to confirm that it is of an acceptable scientific standard.

# Reviewer report: Reviewer response for version 2

**DOI:** 10.21956/wellcomeopenres.20917.r54428

**Published:** 2023-01-25

**Authors:** Chris Keyworth

**Affiliations:** 1 Manchester Centre for Health Psychology, Division of Psychology and Mental Health, University of Manchester, Manchester, UK

I wish to thank the authors for the thoughtful and detailed response to the previous comments. These have all been addressed and I am happy to recommend publication.

Is the work clearly and accurately presented and does it cite the current literature?

Yes

If applicable, is the statistical analysis and its interpretation appropriate?

Not applicable

Are all the source data underlying the results available to ensure full reproducibility?

Partly

Is the study design appropriate and is the work technically sound?

Yes

Are the conclusions drawn adequately supported by the results?

Yes

Are sufficient details of methods and analysis provided to allow replication by others?

Partly

Reviewer Expertise:

NA

I confirm that I have read this submission and believe that I have an appropriate level of expertise to confirm that it is of an acceptable scientific standard.

# Reviewer report: Reviewer response for version 1

**DOI:** 10.21956/wellcomeopenres.19961.r53017

**Published:** 2022-12-22

**Authors:** Stephan Dombrowski

**Affiliations:** 1 Faculty of Kinesiology, University of New Brunswick, Fredericton, NB, Canada

Thanks for the opportunity to review this manuscript. I include some minor comments below for the authors to consider.

 Minor point: “Aberdeen Health Psychology Group, University of Aberdeen, Aberdeen, USA”, p.1 - The Aberdeen referred to here is in Scotland, UK.

 Minor point: “A comprehensive Behaviour Change Intervention Ontology (BCIO) is being developed as part of the Human Behaviour-Change (Michie *et al.*, 2017; Michie *et al., *2021).” – p. 3 should read Human Behaviour Change Project.

 “This enables the answering of questions about the effectiveness of behaviour change interventions and how effects are modified according to different behaviours and contexts (populations and settings), and about the way components of intervention work together to achieve behaviour change.”, p.3 – Why does this sentence include populations and settings in brackets? Are these examples of context components, or related to them in some way?  It would be useful to integrate these fully into the sentence.

 The paper states that the aim is “to gather data with which to update the BCTTv1 and convert it into a BCT ontology.” (p.3). The first aim I can see being met, but there is no conversion into a BCT ontology, which is beyond the scope of the paper. Perhaps rephrase as gathering information which would inform the conversion, or something along those lines?

 The level headings in the methods section are a little confusing to me. Are ‘Design and sample’ a level 2 heading, with ‘Stage one…’, ‘Stage two…’, etc. as level 3 headings underneath it? In general, the information presented in the methods section is more about the information sources than samples. Perhaps the level of the headers can be reviewed and the term ‘sample’ can be reviewed to ensure it is in line with the presented information.

 Under the heading “Stage one: Gathering feedback about the BCTTv1” the authors outline 5 sources of information, but Table 1 lists 6 sources.

 A bit more details as to how relevant papers for new BCTs were identified would add value. For example, did the authors examine all systematic reviews which used BCT v1 to code intervention content for potential proposals of new BCTs and/or amendments to labels and definitions?  This could have been a substantial study in itself.

 How were behaviour classification systems included in the dataset? These provide alternative (albeit often overlapping) techniques and definitions, but are not comments in themselves. Were different techniques which were included in another classification system conceptualised as a comment in the analysis?

 For ease of reading, Table 3 and 4 could be combined in a flowchart, rather than two separate tables. The flowchart could also include the final number of interest (282 comments), which currently only appears in the text.

 Were there no comments at all made to the examples provided alongside the BCT definitions and labels? Having read on, it seems that examples were included as part of this category, as suggested by the sentence “92 comments contained suggestions relating to amending a BCT label, definition or example” (p.7). I would suggest to include examples as part of the category label, as these are important.

 Can the sentences “Additionally, in accordance with ontological best practice, BCTs that refer to two separate techniques within the same definition should be separated out, for example, ‘problem solving’ should be ‘problem solving BCT’ and ‘prompt problem solving BCT’.”(p.7) be clarified?  Does this suggest that using problem solving as a BCT is a separate technique from prompting the use of problem solving?

Is the work clearly and accurately presented and does it cite the current literature?

Yes

If applicable, is the statistical analysis and its interpretation appropriate?

Not applicable

Are all the source data underlying the results available to ensure full reproducibility?

Yes

Is the study design appropriate and is the work technically sound?

Yes

Are the conclusions drawn adequately supported by the results?

Yes

Are sufficient details of methods and analysis provided to allow replication by others?

Partly

Reviewer Expertise:

behaviour change

I confirm that I have read this submission and believe that I have an appropriate level of expertise to confirm that it is of an acceptable scientific standard, however I have significant reservations, as outlined above.

# Response

**Published:** 2023-01-10

**Authors:** Marta Marques

**Affiliations:** Trinity College Dublin, Ireland

Thanks for the opportunity to review this manuscript. I include some minor comments below for the authors to consider.

- *We would like to thank the reviewer for the positive feedback on our manuscript. We have addressed all comments raised.*

Minor point: “Aberdeen Health Psychology Group, University of Aberdeen, Aberdeen, USA”, p.1 - The Aberdeen referred to here is in Scotland, UK.

- *We thank the reviewer for noticing this typo, we have no updated the affiliation to "Scotland, United Kingdom".*

Minor point: “A comprehensive Behaviour Change Intervention Ontology (BCIO) is being developed as part of the Human Behaviour-Change (Michie *et al.*, 2017; Michie *et al., *2021).” – p. 3 should read Human Behaviour Change Project.

- *We have corrected the sentence to include the word project. *

“This enables the answering of questions about the effectiveness of behaviour change interventions and how effects are modified according to different behaviours and contexts (populations and settings), and about the way components of intervention work together to achieve behaviour change.”, p.3 – Why does this sentence include populations and settings in brackets? Are these examples of context components, or related to them in some way?  It would be useful to integrate these fully into the sentence.

- *Thank you for the suggestion. We changed the sentence to: "*This enables the answering of questions about the effectiveness of behaviour change interventions and how effects are modified according to different behaviours and characteristics of the population and setting, and about the way components of intervention work together to achieve behaviour change".

The paper states that the aim is “to gather data with which to update the BCTTv1 and convert it into a BCT ontology.” (p.3). The first aim I can see being met, but there is no conversion into a BCT ontology, which is beyond the scope of the paper. Perhaps rephrase as gathering information which would inform the conversion, or something along those lines?

- *We thank the reviewer for the suggestion and understand the aim could be misleading for the reader as it was stated. We have changed the paragraph as follows: "The* *primary aim of this* *study* *was* *to gather data with which to update the BCTTv1* *.* *This paper reports an analysis of feedback about BCTTv1 from experts and intervention developers from a variety of fields. Feedback was sought on the limitations and* *associated* *improvements that could be made to BCTTv1* *. The later including* *adding BCTs, improving BCT labels and definitions, and changing groupings and structure. Recommendations* *for developing the BCTTv1 into a BCT Ontology* *are made based on the feedback."*

The level headings in the methods section are a little confusing to me. Are ‘Design and sample’ a level 2 heading, with ‘Stage one…’, ‘Stage two…’, etc. as level 3 headings underneath it? In general, the information presented in the methods section is more about the information sources than samples. Perhaps the level of the headers can be reviewed and the term ‘sample’ can be reviewed to ensure it is in line with the presented information.

- *We have changed as suggested by the reviewer.*

Under the heading “Stage one: Gathering feedback about the BCTTv1” the authors outline 5 sources of information, but Table 1 lists 6 sources.

- *In the text we refer to the two surveys only once that is why the numbers do not correspond to the six sources in the table.*

A bit more details as to how relevant papers for new BCTs were identified would add value. For example, did the authors examine all systematic reviews which used BCT v1 to code intervention content for potential proposals of new BCTs and/or amendments to labels and definitions?  This could have been a substantial study in itself.

- *We appreciate the reviewer comment. Research reports were only identified if they explicitly suggested a new BCT/recommendation for revision/new classification system. We did not have the resources to code systematic reviews or interventions.*

How were behaviour classification systems included in the dataset? These provide alternative (albeit often overlapping) techniques and definitions, but are not comments in themselves. Were different techniques which were included in another classification system conceptualised as a comment in the analysis?

- *To clarify this point, we have added the following information: "5) relevant published research reports proposing new BCTs and/or changes to BCTTv1 or other classification systems of BCTs. Definitions for additional BCTs found within other classification systems were added to the dataset verbatim from the relevant publication."*

For ease of reading, Table 3 and 4 could be combined in a flowchart, rather than two separate tables. The flowchart could also include the final number of interest (282 comments), which currently only appears in the text.

- *As suggested, we have now produced a flowchart instead of tables 3 and 4. We agree it is much better for the reader to have this information in a flowchart. Thank you. *

Were there no comments at all made to the examples provided alongside the BCT definitions and labels? Having read on, it seems that examples were included as part of this category, as suggested by the sentence “92 comments contained suggestions relating to amending a BCT label, definition or example” (p.7). I would suggest to include examples as part of the category label, as these are important.

- *We thank the reviewer for this comment. The part "examples" should not be in the text as the decision was to focus on labels and definitions. We have noew changed it. *

Can the sentences “Additionally, in accordance with ontological best practice, BCTs that refer to two separate techniques within the same definition should be separated out, for example, ‘problem solving’ should be ‘problem solving BCT’ and ‘prompt problem solving BCT’.”(p.7) be clarified?  Does this suggest that using problem solving as a BCT is a separate technique from prompting the use of problem solving?

- *Yes, it means they are two separate BCTs. To clarify, we changed the sentence as follows: "Additionally, in accordance with ontological best practice, existing BCTs in the BCTTv1 that refer to more than one technique in their definition should be separated into more than one BCT. For example, the BCT in the BCTTv1 "problem solving" should be "problem solving BCT" (referring to person analysing factors influencing the behaviour and generating and selecting strategies to overcome barriers) and "prompt problem solving BCT" (referring to the source prompting the person to analyse factors influencing the behaviour and generating and selecting strategies to overcome barriers with the person)."*

# Reviewer report: Reviewer response for version 1

**DOI:** 10.21956/wellcomeopenres.19961.r52316

**Published:** 2022-10-11

**Authors:** Chris Keyworth

**Affiliations:** 1 Manchester Centre for Health Psychology, Division of Psychology and Mental Health, University of Manchester, Manchester, UK

Thank you for the invitation to review this paper aiming to gather data to inform the development of BCTTv1 into a more elaborated knowledge structure (an ontology). This is an important piece of work and will be valuable in informing future developments of the BCTT and ontology. I enjoyed reading the paper and can see the value of the work for informing future research, policy and practice. Although the data gathered does not include a systematic search of the literature (which the authors acknowledge), a strength of this work is the triangulation approach, in gathering a wide range of sources and gaining extensive feedback.

 There are some points below which I feel would strengthen the manuscript, particularly around the methods/analysis used to synthesise the data.

- Minor point – abstract – isn’t clear what the final sentence of the results is referring to. Perhaps just a little refinement to say recommendations for improving the taxonomy? The three items presented also don’t make sense in isolation. There are some good examples throughout the paper where this is much clearer (see points below).
- Intro – for the lay reader, can you say what the “16 higher-order groupings” refer to and maybe provide an example? This is a little broad at present. E.g., overarching theme of each group – group 1 refers to goals and planning. I suspect this paper is going to be read very widely read by a number of different audiences, some of whom won’t be familiar with the terminology.
- “The BCTTv1 was developed over an iterative programme of research studies” – would some dates be helpful for historical context? Could use data from Table 1.
- At first mention of the Behaviour Change Wheel Framework – could you very briefly describe the framework and its foundations – i.e., synthesis of 19 frameworks, allows researchers to make recommendations for policy etc.
- “…is being developed as part of the Human Behaviour-Change (Michie et al., 2017; Michie et al., 2021) – I think the word “project” might be missing here?
- Same paragraph, could “source” be changed to “source of the intervention”? Does it also need something like “i.e., who delivers the intervention”? Also perhaps amend to “intervention setting” instead of just “setting” for extra clarity.
- Are there any examples of “artificial-intelligence based methods” that you could cite? Is data mining an example?
- The aims could be a little clearer here and perhaps in the abstract also. For example, there are at least two aims which could be more explicit: 1) to gather data to update BCTTv1 and convert it into a BCT ontology, and 2) provide recommendations for …. (this bit isn’t immediately clear and could be more explicit) – gather data to inform the BCT ontology? Same comment applies to the abstract.
- I really like the first section of the design and sample section – clear and explicitly outlining the main aims of the study. Can this be used to address the point about making the study aims explicit?
- Methods - typo at top of p4 - any use of – should be user.
- Sentence starting “The feedback...” is a tad long and difficult to follow. Could you rework? This should improve the clarity.
- In stage two (the evidence synthesis) there could be more methodological detail added here. What type of coding was done? Did the two coders review each source independently, then agree upon the final code? Any inter-rater reliability? I’m also not sure how the categories were derived. Some clarity in the sentence mentioned above should help.
- Paragraph under stage three is good – I like the clear definitions that are presented.
- The sentence “The number of users that contributed to each feedback source ranged from 22 to 105.” Would be better suited in the paragraph above.
- Under additional BCTs it would be good to know a little more about the 22 additional BCTs. It left me intrigued wondering what the new suggestions were. I understand this will be covered in more detail in the next paper, but is there anything at all you can say about what the extra ones were? Or which higher order groupings they belonged to? It would be interesting to know which if the higher order groupings the suggestions belonged to.
- In the labels or definitions of BCTs, I like the examples that are provided. These will definitely help the lay reader. I was a little unclear what table 5 was referring to? Is this the number of suggestions about amending a BCT label, definition, or adding an example? Or all of them?
- Like the description of Amendments to BCTTv1 groupings. I also like the frequently reported issues with illustrative quotes. Should this section come before “Amendments to BCTTv1 groupings” as the quotes are about labels and definitions?
- The section “Stage three: Recommendations for ontology development” is clear and I like the three clear recommendations.
- Use of BCIO acronym could be consistent throughout the paper – is referred to as “BCI ontology” in the discussion.

Thanks for the opportunity to review this important work. I hope you find the comments useful.

Is the work clearly and accurately presented and does it cite the current literature?

Yes

If applicable, is the statistical analysis and its interpretation appropriate?

Not applicable

Are all the source data underlying the results available to ensure full reproducibility?

Partly

Is the study design appropriate and is the work technically sound?

Yes

Are the conclusions drawn adequately supported by the results?

Yes

Are sufficient details of methods and analysis provided to allow replication by others?

Partly

Reviewer Expertise:

Behaviour change

I confirm that I have read this submission and believe that I have an appropriate level of expertise to confirm that it is of an acceptable scientific standard, however I have significant reservations, as outlined above.

# Response

**Published:** 2023-01-10

**Authors:** Marta Marques

**Affiliations:** Trinity College Dublin, Ireland

Thank you for the invitation to review this paper aiming to gather data to inform the development of BCTTv1 into a more elaborated knowledge structure (an ontology). This is an important piece of work and will be valuable in informing future developments of the BCTT and ontology. I enjoyed reading the paper and can see the value of the work for informing future research, policy and practice. Although the data gathered does not include a systematic search of the literature (which the authors acknowledge), a strength of this work is the triangulation approach, in gathering a wide range of sources and gaining extensive feedback. *We thank the reviewer for the positive feedback about this paper. All comments made by the reviewer were addressed.*

 There are some points below which I feel would strengthen the manuscript, particularly around the methods/analysis used to synthesise the data.

- Minor point – abstract – isn’t clear what the final sentence of the results is referring to. Perhaps just a little refinement to say recommendations for improving the taxonomy? The three items presented also don’t make sense in isolation. There are some good examples throughout the paper where this is much clearer (see points below).

*As suggested, we changed the sentence as following: "Three recommendations to improve the BCTTv1 resulted from this analysis: to review the label and definition of each BCT, the 16 groupings of BCTs, and the examples illustrating BCTs.*

- Intro – for the lay reader, can you say what the “16 higher-order groupings” refer to and maybe provide an example? This is a little broad at present. E.g., overarching theme of each group – group 1 refers to goals and planning. I suspect this paper is going to be read very widely read by a number of different audiences, some of whom won’t be familiar with the terminology.

For ease of reading and as suggested by the reviewer. we have changed the sentence to: "The Behaviour Change Techniques Taxonomy v1 (BCTT v1) ( Michie *et al.,* 2013) is the most widely used classification of BCTs. The BCTTv1 provides a list of 93 clearly labelled and defined BCTs, organised in 16 higher-order groupings representing the function of the BCTs in each group, e.g. group 1 refers to goals and planning BCTs.

- “The BCTTv1 was developed over an iterative programme of research studies” – would some dates be helpful for historical context? Could use data from Table 1.

Table 1 refers to the feedback of the BCTTv1 that was conducted as part of the current study, while the sentence mentioned refers to the process of the development of the BCTTv1 which is described in detail in the reference now provided (Michie et al., 2015). We hope this is clearer now.

- At first mention of the Behaviour Change Wheel Framework – could you very briefly describe the framework and its foundations – i.e., synthesis of 19 frameworks, allows researchers to make recommendations for policy etc.

*As suggested, we have added a sentence briefly describing the framework, as follows: "The Behaviour Change Wheel* *is an integrative framework that derived from a synthesis of 19 frameworks to aid the systematic development and evaluation of behaviour change interventions* *and has been used across a wide range of behaviours and to inform policy (West et al., 2020). The framework provides a systematic way of identifying i. the problem in behavioural terms, ii. what needs to change for the target behaviour to change in terms of capabilities, opportunities and motivation (COM-B model), iii. the relevant intervention functions and techniques to change the target behaviour, and iv. the relevant policy categories (societal or organizational strategies) for implementing sustained behavioural changes."*

- “…is being developed as part of the Human Behaviour-Change (Michie et al., 2017; Michie et al., 2021) – I think the word “project” might be missing here?

*We thank the reviewer for noticing this. We have added the word "project".*

  

- Same paragraph, could “source” be changed to “source of the intervention”? Does it also need something like “i.e., who delivers the intervention”? Also perhaps amend to “intervention setting” instead of just “setting” for extra clarity.

*As recommended by the reviewer we have changed the sentence as follows: "* *The BCIO consists of an upper level with 42 entities, specifying features of behaviour change interventions, such as mode of delivery (* *Marques et al., 2021* *), source of the intervention (* *Norris et al., 2021* *), and the setting where the intervention takes place (* *Norris et al., 2020* *). One of the entities in the BCIO is BCT , specified as part of the content in a given behaviour change intervention scenario (* *Michie et al., 2021* *)."*

- Are there any examples of “artificial-intelligence based methods” that you could cite? Is data mining an example?

*We added two examples between brackets "(e.g. natural language processing, machine learning)"*

- The aims could be a little clearer here and perhaps in the abstract also. For example, there are at least two aims which could be more explicit: 1) to gather data to update BCTTv1 and convert it into a BCT ontology, and 2) provide recommendations for …. (this bit isn’t immediately clear and could be more explicit) – gather data to inform the BCT ontology? Same comment applies to the abstract.

*Reviewer 2 also suggested changes to the aims, namely that the recommendations for its development into an ontology should be a secondary aim. We have therefore revised that section as follows:* *The primary aim of this study was to gather data with which to update the BCTTv1. This paper reports an analysis of feedback about BCTTv1 from experts and intervention developers from a variety of fields. Feedback was sought on the limitations and associated improvements that could be made to BCTTv1. The latter included in relation to adding BCTs, improving BCT labels and definitions, and changing groupings and structure. Recommendations for developing the BCTTv1 into a BCT Ontology are made based on the feedback."* *We also changed the abstract to:" This study aimed to gather data to improve the BCTTv1 and provide recommendations for developing it into a more elaborated knowledge structure, an ontology."*

- I really like the first section of the design and sample section – clear and explicitly outlining the main aims of the study. Can this be used to address the point about making the study aims explicit?

*We thank the reviewer for the suggestion. We have already improved the aims as explained in the previous comments.*

- Methods - typo at top of p4 - any use of – should be user.

*Thank you for noticing the typo, we have amended it.*

- Sentence starting “The feedback...” is a tad long and difficult to follow. Could you rework? This should improve the clarity.

*We have revised as follows: "* *Data from each source of feedback relating to the BCTTv1 were reviewed by two authors (EC and MM)* *. A content analysis was performed on the data collected from the BCTTv1 user survey (EC). Four main categories were formed based on the main issues raised:* *additional BCTs, amendments to labels or definitions of BCTs, amendments to the BCT groupings, and general improvements. Two authors (EC and MM) used these categories to code the remaining sources of feedback jointly (via online meetings). As this process was undertaken jointly, no interrater reliability data was produced".*

- In stage two (the evidence synthesis) there could be more methodological detail added here. What type of coding was done? Did the two coders review each source independently, then agree upon the final code? Any inter-rater reliability? I’m also not sure how the categories were derived. Some clarity in the sentence mentioned above should help.

*We have provided more methodological detail in this section as follows: "Data from each source of feedback relating to the BCTTv1 were reviewed by two authors (EC and MM)* *. A content analysis was performed on the data collected from the BCTTv1 user survey (EC). Four main categories were formed based on the main issues raised:* *additional BCTs, amendments to labels or definitions of BCTs, amendments to the BCT groupings, and general improvements. Two authors (EC and MM) used these categories to code the remaining sources of feedback jointly (via online meetings). As this process was undertaken jointly, no interrater reliability data was produced".*

- Paragraph under stage three is good – I like the clear definitions that are presented.

*Thank you for the positive feedback.*

  

- The sentence “The number of users that contributed to each feedback source ranged from 22 to 105.” Would be better suited in the paragraph above.

*We agree, and have moved the sentence to the previous paragraph. *

- Under additional BCTs it would be good to know a little more about the 22 additional BCTs. It left me intrigued wondering what the new suggestions were. I understand this will be covered in more detail in the next paper, but is there anything at all you can say about what the extra ones were? Or which higher order groupings they belonged to? It would be interesting to know which if the higher order groupings the suggestions belonged to.

*Review by the research team resulted in 22 suggestions for new BCTs. These suggestions, along with corresponding relationships are to be further developed in an ontology of BCTs.*

- In the labels or definitions of BCTs, I like the examples that are provided. These will definitely help the lay reader. I was a little unclear what table 5 was referring to? Is this the number of suggestions about amending a BCT label, definition, or adding an example? Or all of them?

*We are glad the examples were considered helpful. Table 5 refers to number of suggestions to amend either the BCT label, definition or example as stated in the text. We have changed the title of the table to make it clearer, as follows: "Number of suggestions made related to the label, definition or example of specific behaviour change techniques (BCTs)."*

- Like the description of Amendments to BCTTv1 groupings. I also like the frequently reported issues with illustrative quotes. Should this section come before “Amendments to BCTTv1 groupings” as the quotes are about labels and definitions?

*We have changed the order as suggested by the reviewer.*

- The section “Stage three: Recommendations for ontology development” is clear and I like the three clear recommendations.

*Thank you for the positive feedback.*

- Use of BCIO acronym could be consistent throughout the paper – is referred to as “BCI ontology” in the discussion.

We have revised and amended throughout the text. Thanks for the opportunity to review this important work. I hope you find the comments useful. *The comments were really hepful. Thank you for the reiview*
